# Analyzing maternal mortality rate in rural China by Grey-Markov model

**DOI:** 10.1097/MD.0000000000014384

**Published:** 2019-02-08

**Authors:** Yawen Wang, Zhongzhou Shen, Yu Jiang

**Affiliations:** School of Public Health, Chinese Academy of Medical Sciences/Peking Union Medical College, Beijing, China.

**Keywords:** grey model, Markov, maternal mortality rate, prediction, rural

## Abstract

Maternal mortality rate (MMR) in China has reduced during a decade but still higher than many countries around the world. Rural China is the key region which affects over all maternal death. This study aims to develop a suitable model in forecasting rural MMR and offer some suggestions for rural MMR intervention. Data in this study were collected through the Health Statistical Yearbook (2017) which included the overall MMR in China and urban and rural mortality rate. A basic grey model (GM(1,1)), 3 metabolic grey models (MGM), and a hybrid GM(1,1)–Markov model were presented to estimate rural MMR tendency. Average relative error (ARE), the post-test ratio (*C*), and small error probability (*P*) were adopted to evaluate models’ fitting performance while forecasting effectiveness was compared by relative error.

The MMR in rural China reduced obviously from 63.0 per 100,000 live births in 2005 to 21.1 per 100,000 live births in 2017. One basic GM(1,1) model was built to fit the rural MMR and the expression was *X*^((1)) (*k* + 1) = 553.80e^0.0947*k* – 550.00 (*C* = 0.0456, *P* > .99). Three MGM models expressions were *X*^((1)) (*k* + 1)  = 548.67e^0.0923*k* – 503.17 (*C* = 0.0540, *P* > .99), *X*^((1)) (*k* + 1) = 449.39e^0.0887*k* – 408.09 (*C* = 0.0560, *P* > .99), *X*^((1)) (*k* + 1) = 461.33e^0.0893*k* – 425.23(*C* = 0.0660, *P* > .99). Hybrid GM(1,1)–Markov model showed the best fitting performance (*C* = 0.0804, *P* > .99). The relative errors of basic GM(1,1) model and hybrid model in fitting part were 2.42% and 2.03%, respectively, while 5.35% and 2.08%, respectively, in forecasting part. The average relative errors of MGM were 2.07% in fitting part and 17.37% in forecasting part.

Data update was crucial in maintain model's effectiveness. The hybrid GM(1,1)–Markov model was better than basic GM(1,1) model in rural MMR prediction. It could be considered as a decision-making tool in rural MMR intervention.

## 1 Introduction

In 2000, the United Nations Millennium Development Goals (MDGs) had declared reducing child mortality and improving maternal health as a global aim, including MDG 5 which called for a reduction of maternal mortality rate (MMR) between 1990 and 2015.^[1]^ In 2015, the World Health Organization (WHO) came up with “Strategies toward ending preventable maternal mortality (EPMM)” (EPMM Strategies), which emphasized the importance of maternal mortality reduction in sustainable development goal (SDG) period.^[2]^ Even with the help of international organizations and local governments, some reports indicated that the MMR reduction progress was much slower than MDG 5 requirement.^[3]^ Much more efficient interventions are needed.

China became a signatory of MDGs in September 2000 and in the meantime, children and maternal health became an important part of Healthy China 2030 Planning Outline. The overall MMR have declined to 19.6 per 100,000 livebirths in 2017 from 53.0 per 100,000 livebirths in 2000. It seems a great achievement, but large population base means there still has >30,000 maternal death because of various obstetric disadvantage outcomes. The distribution of MMR in China has notable region features.^[4]^ Some studies showed that the north China has higher MMR and under-5 child mortality rate (U5MR) than south, western regions showed higher MMR and U5MR than eastern regions.^[5]^ Besides, an obvious urban–rural difference can be seen on child mortality and maternal mortality due to medical condition and social economic differences. Rapidly decrease of rural MMR contributed to lower overall MMR but it still serious comparing with other developed countries.

Time series prediction are indicated useful in disease prevention. Linear regression, time series analysis, and neural network model are most commonly used. The grey model (GM) is populated thanks to its small sample size and uncertain information recognition. Basically, all data can be divided into 3 classes, white, black, and grey systems.^[6]^ According to the information we know about the data, black system refers to uninformed data, neither the certain problem nor the data characteristic. White system indicates all-knowing data while grey system means uncertain problems, incomplete information. In MMR prediction, all the information we know are time and incidence rate. Basic GM(1,1) model means first order equation and single variable^[7]^ and was adopted in many real-word researches.^[8,9]^ This model prefer sequence with exponential tendency and series with fluctuation may decrease the model's performance.^[10]^ Thus more models should be adopted to choose a better one.

Markov chain model is widely used in cost-effectiveness analysis.^[11–13]^ This model is a dynamic system which based on the state transition.^[14]^ The system's state is randomized at all time and independent with prior states, this characteristic is called non-aftereffect property or Markov process. State transition probability matrix is the model's foundation. The advantage of Markov chain is learning and predicting the fluctuation and improving predicting performance. Some researches combined GM and Markov in fitting and forecasting health economic data or engineer problems and got high accuracy.^[15,16]^ Since there are less applications in medical related research, the hybrid model's performance in this field is unclear.

In this study, basic GM(1,1) model, 3 MGM models and hybrid GM(1,1)–Markov model were built to fit and predict MMR in rural China and evaluated their performances. According to the MMR condition, we came up with some advises in rural maternal mortality intervention.

## 2 Materials and methods

### 2.1 Materials source

The yearly incidence data of MMR in China from 2005 to 2017 were collected from the Health Statistical Yearbook, which reflects the health care development of China and health status of residents lived in 31 provinces in mainland China and published by the National Health Commission of China. The sample size of GM (1,1) model was 10 since the model has less requirement of data. The basic model was built with data from 2005 to 2014 so that the last MGM could be built with MMR in 2017. If basic model was built with values from 2005 to 2017, then no data were available to build MGM. Three metabolic models were built with actual data and data forecasted by prior models. GM-Markov model was built with MMR from 2005 to 2014.

### 2.2 Basic GM(1,1) model

The steps of building a GM (1,1) model include original time sequence, accumulated generating operation (AGO), adjacent neighbor means, whitenization equation, and inverse AGO.

The nonnegative original time sequence 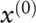 and AGO time series 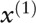 showed as:  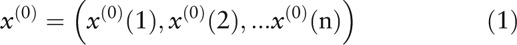



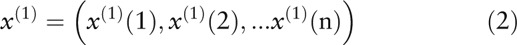


n is the sample size of the data.

Adjacent neighbor means. Calculating the mean of AGO time series and showed as: 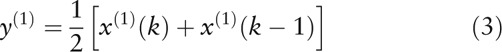


*k* = 2,3…,n.

The whitenization equation was showed as: 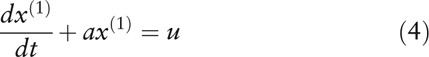


In this equation, *a* is developing coefficient and *u* is control variable. These are 2 parameters of GM(1,1) model. In addition, *a* is an assistant to estimate the GM(1,1) model's prediction length (Table 1).

**Table 1:**
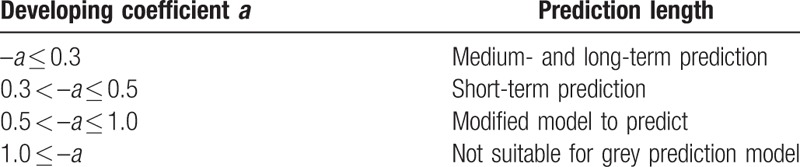
Developing coefficient and prediction length.

Inverse AGO was done to develop GM(1,1) model and showed as: 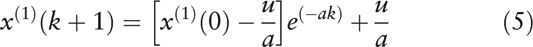


### 2.3 MGM models

Data update is the model's characteristic. One old data is excluded and a new data is adopted to develop a more accurate model or maintain the model's performance. This process can be shown by followed equations.

The original model shown as equation (1).

If *x*(n+1) is the most recent data, it will take the place of 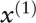 and the new model is built with different sequence which shows as: 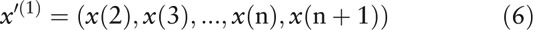


This is called first-step metabolic model. Second-step metabolic model can be developed with data forecasted by first-step metabolic model and the rest can be done in the same manner.

### 2.4 GM(1,1)–Markov chain

Step 1: The partition of transferring

The actual incidence of MMR in China from 2005 to 2014 and the data forecasted by basic GM(1,1) model are known and the relative error is obtained. The relative error of fitted values can be divided into >3 different status showed as: 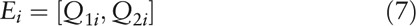


where *i* = 1, 2, …, n.

Step 2: The establishment of the state transition probability matrix

If *p*_*ij*_(*m*) means the probability of the relative error transferring from state *i* to *j* in step *m*, the Markov state transaction probability matrix consisted of *p*_*ij*_ (*m*) can be presented as: 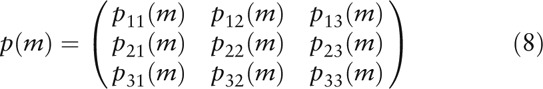


and 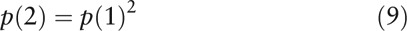


Step3: Markov property test

Chi-squire test is adopted to test Markov property. 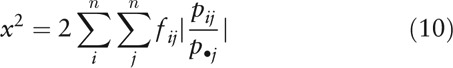



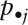
 is the marginal probability of *j* row.

Data forecasted by GM(1,1) model meet the requirement of Markov property if 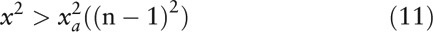


n is the number of status. If this inequality cannot be satisfied, the series is not suitable for Markov process.

Step 4: Revision of GM(1,1) model

According to Markov state transition probability matrix, each relative value belongs to a status [*Q*1, *Q*2], the grey model is revised by 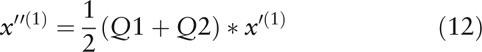


### 2.5 Model test

#### 2.5.1 Relative error

The relative error of an optimal model should <5% generally, but it is still acceptable if the relative error is >5% but <20%.

#### 2.5.2 The post-test ratio (*C*)

*C* = Se/Sx. Se means the standard deviation of residual series and Sx means the standard deviation of original time series. The value reflects the concentration degree of the difference between predicted value and actual value. Smaller *C* means more concentrated difference.

#### 2.5.3 Small error probability (*P*)

Calculating the difference between residual and it's mean and *P* is the ratio of the difference to 0.6475Sx. Greater *P* means closer difference to 0.6475Sx. *P* and *C* are combined to evaluate the fitting effect of GM(1,1) model (Table 2).

**Table 2:**
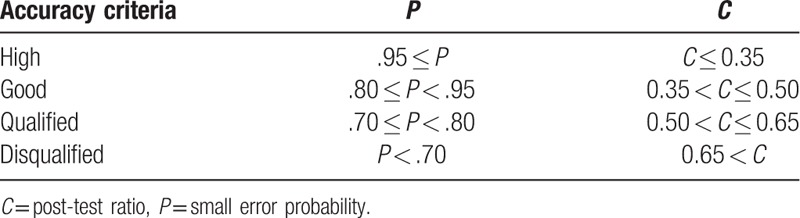
Accuracy evaluation criteria of GM(1,1) model.

### 2.6 Data processing and analysis

Excel 2016 was used to build the database of MMR in rural China and R 3.4.3 software was adopted to develop the basic GM(1,1) model. Significant level is .05.

## 3 Results

The MMR in rural China from 2005 to 2017 showed an obviously downward trend but still severe than urban and overall MMR. The MMR in rural China was 63.0 per 100,000 livebirths in 2005 and came to the lowest in 2016 with 20.0 per 100,000 livebirths. It had a slightly increase in the past year while the incidence rate was 21.1 per 100,000 livebirths.

### 3.1 Basic GM(1,1) model

The basic GM(1,1) model was built with data from 2005 to 2014 and the expression was *x*^(1)^(*k*+1) = 553.80*e*^0.0947*k*^ − 550.00 (–*a* = –0.0947, *u* = 52.0795). The post-test ratio (*C*) and small error probability (*P*) were .0456 and .99, respectively. The relative error between actual value and fitting value was 2.42%, which means the model could fit the incidence of MMR in rural China well. The rural MMR in 2015 was forecasted by the basic model and the relative error of forecasting was 5.35%.

### 3.2 Metabolic GM model

The MMR in 2015 was forecasted by basic GM(1,1) model and then it was adopted to build the first-step MGM, the value of 2005 was excluded in the meantime. The incidence of 2016 was predicted by the first-step MGM and adopted to build the second-step MGM. Similarly, the third-step MGM was built. Three MGM models’ expressions were *x*^(1)^(*k*+1) = 548.67*e*^*0.0923k*^ − 503.17 (*C* = 0.0540, *P* > .99), *x*^(1)^(*k* + 1) = 449.39*e*^*0.0887k*^ − 186,408.09 (*C* = 0.0560, *P* > .99), *x*^(1)^(*k* + 1) = 461.33*e*^*0.0893k*^ − 425.23 (*C* = *0.0660*, *P* > .99). Since the MMR in rural China in 2018 is unknown now, the relative error of forecasting of third-step MGM in unknown.

It can be found in Table 3 that the fitting performance of data renewal models were better than basic GM(1,1) model even with a slightly increase of *C*. With the help of new data, the relative error declined. However, the predicting performance of MGM was worse than basic GM(1,1) model.

**Table 3:**
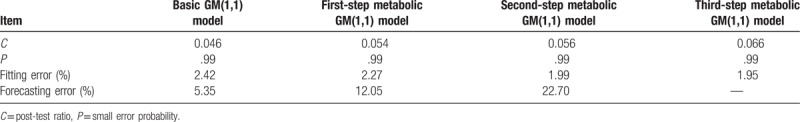
Comparison of 4 models.

### 3.3 Hybrid GM (1,1)–Markov model

The hybrid model was built on the basis of basic GM(1,1) model and the relative error between actual value and fitted value was divided into 3 status according to experience of researchers, which were E1:[0.9491,0.9919], E2:[0.9919,1.0133], and E3:[1.0133,1.0561]. The status of each year was showed at Table 4.

**Table 4:**
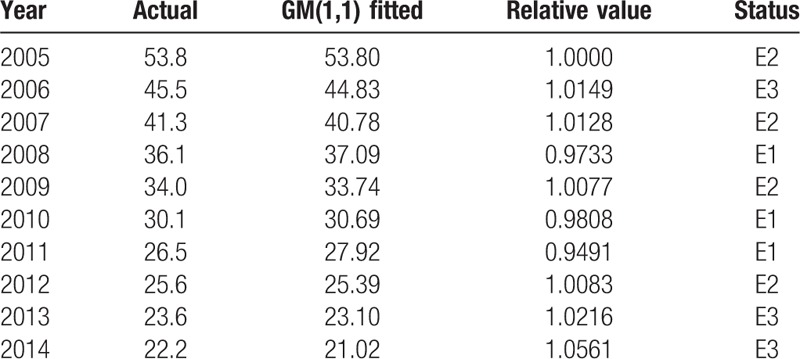
The status of each year.

Thus the Markov state transaction probability matrix was shown as: 
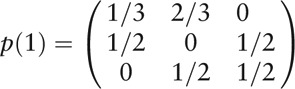


The marginal probabilities were 3/10, 4/10, and 3/10, initialization vectors were 3/10, 4/10, and 3/10 too. Markov property test showed that the time series was suitable to build Markov model. 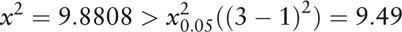


The MMR in rural China in 2015 was calculated by the 3-step state transition probability matrix. Three most recent values were adopted and calculated the forecasted value with different transfer steps. The results were showed at Table 5.

**Table 5:**
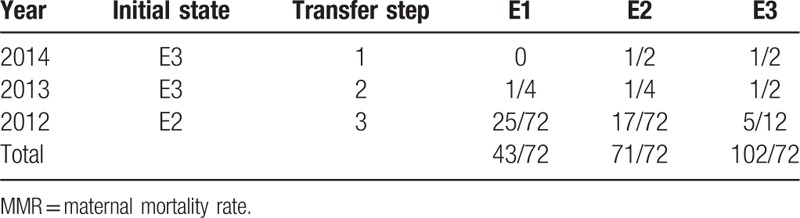
The forecasted state of MMR in rural China in 2015.

According to Table 5, the MMR in rural China in 2015 was most likely to be in E3. Thus the revised GM(1,1)–Markov chain value was *x*″^(1)^ = 0.5∗(1.0133 + 1.0561)∗19.12 = 19.78. The relative error was 2.08%. Values from 2005 to 2014 were fitted by the hybrid model the model had an average relative error by 2.03%, *C* = 0.0804, *P* > .99.

**Figure 1:**
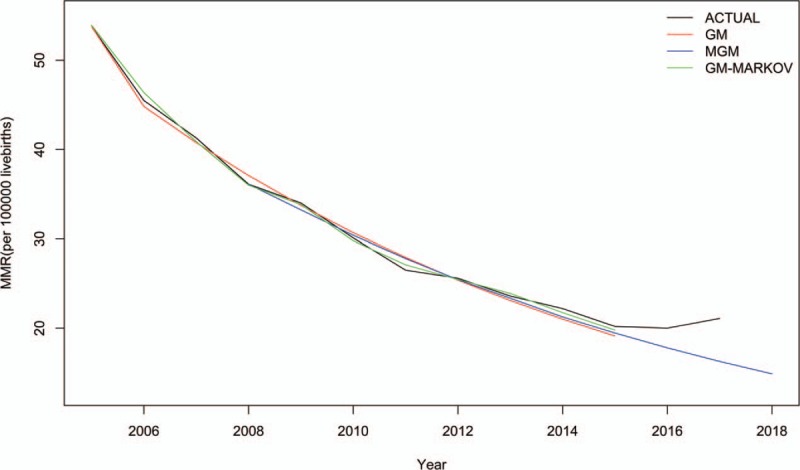
The curves of 3 models and the actual MMR series. MMR = maternal mortality rate.

The original sequence and series fitted by basic GM (1,1) model, third-step MGM and hybrid GM(1,1)–Markov model were shown in Fig. 1. The last value of each curve is predicted data and the rest is fitting data. It can be seen that green line fits black line most both in fitting and forecasting part, which means hybrid model was best for MMR prediction.

## 4 Discussion

The MMR in rural China from 2005 to 2017 showed an obverse reduction. The basic GM(1,1) model showed a well performance in fitting and forecasting. Metabolic models resulted in a better performance with lower average relative error in fitting part. The hybrid GM(1,1)–Markov model can fit the linear and non-linear part of original sequence better than basic grey model, it could be considered as a potential decision-making tool in MMR intervention.

Data update is a basic requirement to maintain model's forecasting performance.^[17]^ With social developing, some new interference factors may act on series develop tendency and old values will be meaningless. Adopting most recent elements and removing old elements to maintain or improve GM(1,1) model's accuracy is common.^[18]^ Three different models were built and the relative error of fitting part decreased with the help of new data. Almost all time series prediction models’ research articles mentioned the time-sensitive, which means the research results were applicable in short order.^[19,20]^ Many unmeasurable influence factors are uncertain in a long term. It was confirmed that the only information about MMR we could know was time, some other factors like medical condition and social economic were represented by time. To minimize forecasting error, the data must contain the most recent develop tendency. It is useful to renew the model by adding forecasted data or actual data and abandoning old one and deliver a mid-and-long term prediction. However, the forecasting performance of MGM went down while most recent predicted values were enrolled. MMR in rural China showed fluctuation during the study period and this may reduce forecasting accuracy of MGM.

GM(1,1)–Markov model showed better performance and the forecasted results may help in health administration. Since grey model fits exponential sequences well, Markov chain could handle with fluctuation.^[21]^ In this study, the average relative error of hybrid model was lower than basic GM(1,1) model in fitting and forecasting part. Markov model makes prediction on the basis of interval and improves prediction accuracy in spite of reduced precision. Generally, since the variates in this study were MMR and time, the results of model only give health department references that it's a time to take some more targeted interventions.

Here still some factors delayed the progress of MMR reduction. Some researches showed that 4 factors were abused for high MMR in rural China.^[22]^ In rural, lack of knowledge and information make it hard to seek help. Besides, the economic condition is a vital factor affects decision to seek help. Disease treatment leads to poverty and poverty makes people more vulnerable. On the other side, once patient decided to go to hospital, the road conditions and vehicles in rural does not seem optimistic. Some researches showed that adverse personal experience and other social determinates of health have link with chronic health problems, which might same with rural women in China.^[23]^ All these disadvantage infectors are adverse to rural maternal mortality reduction and more efficient intervention is required.

To reduce MMR more effectively, some key points should be considered. As reported, lack of knowledge plays an important role in maternal health.^[24]^ Education is the most cost-effectiveness method to improve maternal awareness of seeking professional obstetric support.^[25,26]^ Besides, community-based intervention was suggested to minimize urban-rural difference.^[27,28]^ Primary care at hospital and emergency care accessibility is proved to be useful.^[29]^ Another point needs to be considered is that there is spatial correlation between different regions, areas with high MMR could affect surroundings.^[30]^ This indicates that maternal mortality intervention should focus more on high MMR regions, which has positive effect on its surroundings. Since China has applied national Essential Medicines List in 2009 and required zero drug profit in public hospital in 2017, some essential medicines in primary health care institutions are affordable.^[31]^ There is no doubt the rural MMR will reduce in the next few years.

Here are some limitations in this study. Firstly, the incidence data were unstable because of the geographical variation. In recent years, some rural areas were changed to urban areas according to the new policy. This transition may have no effect at overall MMR, but MMR of rural and urban areas might be affected. Thus the model developed in this study can only give a reference in current maternal mortality intervention. Another shortcoming was the values we collected may differ from actual MMR. Since poverty and inconvenience make rural women hesitate in seeking obstetric support, some maternal death and disadvantage outcomes might miss. Anyway, this study showed a reference in rural MMR prediction, more accurate methods need further discussion.

## Acknowledgments

The authors would like to express their gratitude to anonymous peer reviewers for carefully revising the manuscript and for their useful comments.

## Author contributions

**Conceptualization:** Yawen Wang, Yu Jiang.

**Data curation:** Zhongzhou Shen.

**Formal analysis:** Yawen Wang, Zhongzhou Shen.

**Writing – original draft:** Yawen Wang.

**Writing – review & editing:** Yawen Wang, Zhongzhou Shen, Yu Jiang.

Ya-wen Wang orcid: 0000-0002-1306-8931.
